# Surgical Treatment of Comminuted Coronal Shear Fracture of Distal Humerus

**DOI:** 10.1111/os.12765

**Published:** 2020-09-26

**Authors:** Lin Teng, Gang Zhong

**Affiliations:** ^1^ Department of Orthopedics The First People's Hospital in Shuangliu District/West China Airport Hospital, Sichuan University Chengdu China; ^2^ Department of Orthopedics West China Hospital, Sichuan University Chengdu China

**Keywords:** Capitellum, Distal humeral fracture, Internal fixation, Trochlea

## Abstract

**Objective:**

To investigate the surgical approach, fixation, and clinical effect of comminuted coronal shear fracture of distal humerus.

**Methods:**

From March 2017 to February 2019, we had used open reduction and internal fixation to treat 19 cases of comminuted distal humeral coronal shear fracture. There were 8 males and 11 females, with an average age of 44.6 years (19–72 years). There were 10 cases on left side and 9 cases on right side. All cases were closed fractures. According to Dubberley's classification, there were six cases of type 1, six cases of type 2, and seven cases of type 3. The lateral Kocher approach, extended Kocher approach, extended Kocher approach combined with a medial incision and the olecranon osteotomy approach were used for exposure. Headless screw, Kirschner wire, and suture were used to fix the fractures. Two cases were fixed with hinged elbow fixators additionally. The follow‐up was evaluated by Mayo Elbow Performance Score (MEPS).

**Results:**

All patients were followed up for an average of 17.1 months (range, 12 to 30 months). The average time of fracture union was 8.8 ± 1.9 weeks. There were three cases of degenerative osteoarthritis of elbow and one case of heterotopic ossification after operation. A total of 10 patients underwent removal of implants. At the last follow‐up, the elbow flexion‐extension arc was 130.5° ± 10.5°. The forearm rotation arc was 167.4° ± 6.1°. The MEPS was 85.8 ± 8.5, the results were classified as excellent in nine cases, good in eight, and fair in two. The excellent and good rate was 89.5%. The time of fracture union of type 1 was shorter than type 3 (*P* = 0.024), the elbow flexion‐ extension arc of type 1 fracture was better than type 2 (*P* = 0.043) and type 3 (*P* = 0.012), the forearm rotation arc of type 1 fracture was better than type 3 (*P* = 0.006), the MEPS of type 1 fracture was better than type 2 (*P* = 0.009) and type 3 (*P* = 0.002).

**Conclusion:**

Open reduction and internal fixation with headless screw, Kirschner wire, and suture can be used for the treatment of comminuted distal humeral coronal shear fractures. The elbow joint function can be restored satisfactorily.

## Graphical abstract

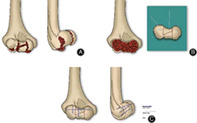

Experience of 19 comminuted distal humeral coronal shear fractures who received open reduction and internal fixation were present in this article. ORIF with headless screw, Kirschner wire, and suture are helpful for anatomical reduction of fracture and restoration of joint integrity.

## Introduction

Distal humeral coronal plane shear fractures include capitellum and trochlear fractures, which are uncommon, accounting for 6% of all distal humeral fractures and 1% of all elbow fractures^1^, ^2^. The mechanism of this injury is that the force is transmitted from the radial head to the capitellum when the elbow is outstretched, or it can also be caused by the posterolateral dislocation of the elbow, the shear force on the articular of distal humerus from the reduction of radial head. This fracture is often combined with radial head fracture, injury of the lateral collateral ligamentous complex, and the avulsion fracture of lateral epicondyle of humerus.

Because of the rotation and separation of the subchondral fragments of capitellum and trochlea, the standard lateral view of the elbow can depict a typical “double arc” sign in some cases^3^. Computer tomography (CT) and three‐dimensional reconstruction can completely show the location and quantity of the fractures, the range of involvement, displacement, concomitant injuries, which are helpful to make a correct preoperative plan^4^, ^5^, ^6^.

Displaced fractures of capitellum and trochlear usually need surgical treatment. Good clinical result requires anatomical reduction of articular fracture and rigid fixation to start functional exercise as soon as possible to avoid a stiff elbow. The most commonly preferred approaches were lateral approach and posterior approach. Lateral approach is sometimes insufficient to expose many comminuted fractures involving trochlear, and posterior olecranon osteotomy may have some complications such as nonunion of the osteotomy site and implant irritation. Internal fixation, excision, arthroscopic operation, and prosthetic replacement could be chosen according to the complexity and condition of the fracture, stability of the reduction, the patient's age and requirements. Open reduction and internal fixation usually was reviewed the recommended treatment for displaced comminuted distal humeral coronal shear fractures. Anti‐glade plate, headless screw, lag screw, Kirschner wire, and suture can be utilized to fix these fractures. But malreduction and poor fixation still can cause elbow dysfunction. Some complications such as nonunion, degenerative arthritis, heterotopic ossification, avascular necrosis, and stiff elbow had been reported previously^4^, ^5^, ^7^, ^8^.

In this study, 19 cases of comminuted distal humeral coronal shear fractures that received open reduction and internal fixation were retrospectively reviewed. Our aims were: (i) to evaluate the internal fixation in comminuted coronal shear fracture of distal humerus; (ii) to investigate the surgical approach of distal humeral coronal shear fracture; (iii) to analyse the clinical outcomes of these patients in the follow‐up and compare the clinical outcomes between different types of fracture.

## Methods

### *Subjects*

The inclusion criteria for enrolling patients were as follows: (i) distal humeral coronal plane shear fractures, including capitellum fractures and/or trochlear fractures; (ii) accepted the treatment of open reduction and internal fixation; (iii) functional exercise and regular follow‐up according to the doctor's acquirement. In contrast, the exclusion criteria were as follows: (i) the distal humeral coronal plane shear fractures were treated by closed reduction, excision, and prosthetic replacement; (ii) combined with elbow rheumatoid arthritis, osteoarthritis, severe osteoporosis, and other diseases which would affect elbow function.

**Fig 1:**
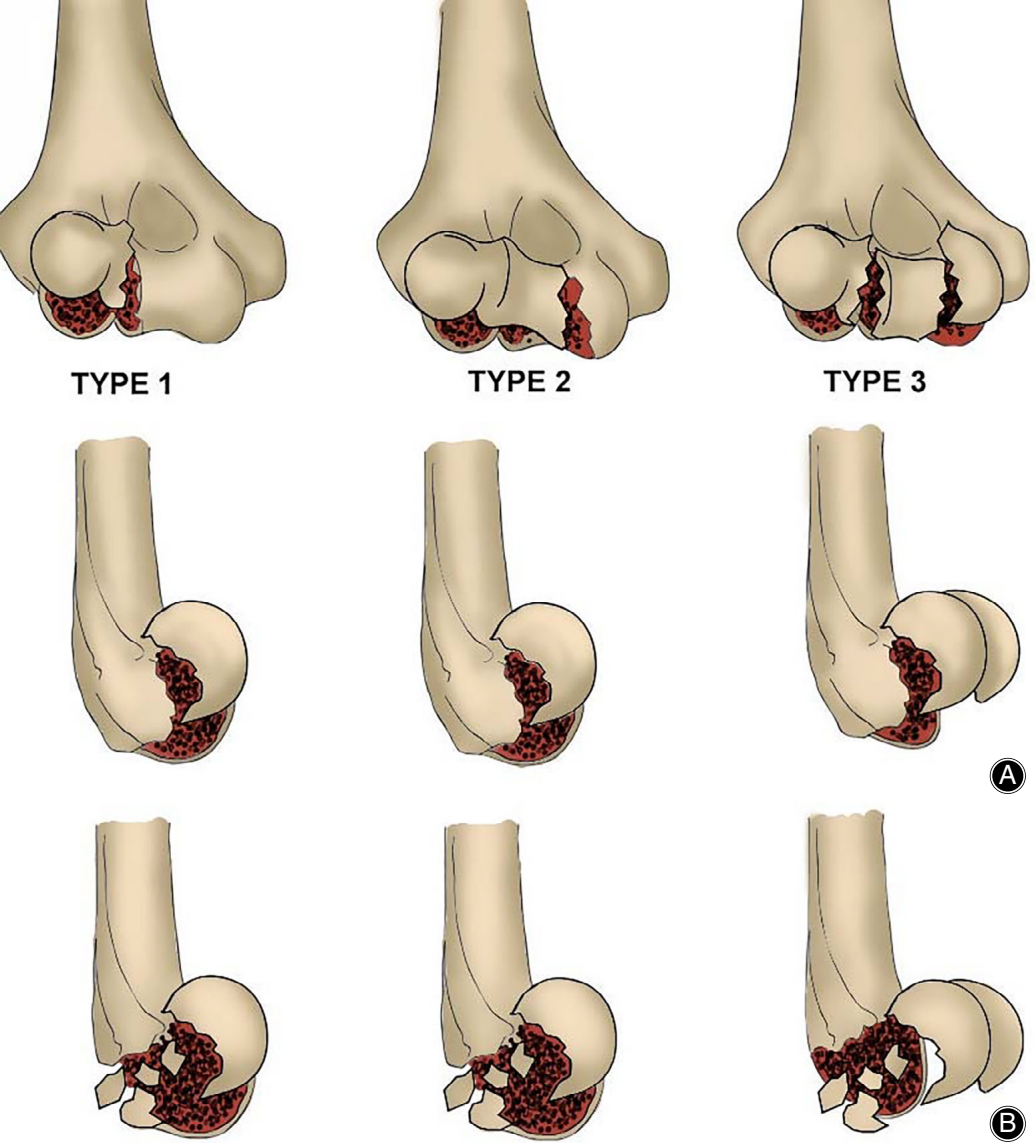
Dubberley's classification of capitellar and/or trochlear fractures. Type 1: Fractures that involve the capitellum with or without lateral trochlear ridge. Type2: Fractures that involve the capitellum and the trochlea as one piece. Type 3: Fractures of both the capitellum and trochlea as separate fragments. Type A: Posterior condylar comminuted absent. Type B: Posterior condylar comminuted present.

A retrospective review of patient files and operation logs between March 2017 to February 2019 was done. In this period, we treated 19 patients who had capitellum and trochlea fractures by open reduction and internal fixation. There were 8 males and 11 females, with an average age of 44.6 years (range, 19 to 72 years). There were 10 patients on the left side and 9 patients on the right side. 2 patients had fractures after falling from height, 12 after falling on flat ground, and 5 patients caused by traffic accidents. According to Dubberley's classification (Fig. 1), six fractures were type 1 (four type 1A, two type 1B), six were type 2 (three type 2A, three type 2B), and seven were type 3 (five type 3A, two type 3B)^9^. Two patients associated with radial head fractures, five associated with lateral collateral ligament injury, one associated with an avulsion fracture of the lateral epicondyle of humerus, and one associated with the medial epicondyle of humerus fracture. This study was approved by the hospital ethics committee, and all patients signed the ethical informed consent after admission.

### *Preoperative Management*

Plaster fixation of the elbow was used after admission. X‐ray film is essential. Computer tomography scan with sagittal and coronal plane reconstruction are recommended to define the fracture and make preoperative plan. Check the stability of the elbow carefully, pay special attention to the lesion of medial or lateral collateral ligaments, such as ecchymosis and valgus instability of the elbow. MRI is examined if necessary. The average time from injury to operation was 5.2 days (range, 2 to10 days).

### *Surgical Procedures*

#### *Approach*

All patients were managed on under general anesthesia. The patients were supine with the upper limb abducting on the operating table. Pneumatic tourniquets were used. A lateral approach included Kocher exposure in four cases and extended Kocher exposure in seven cases was used^10^. When the medial fragment of the trochlea could not be adequately visualized from a lateral approach or combined with medial epicondyle of humerus fracture, further exposure was achieved with a medial approach (six cases) or an olecranon osteotomy (two cases). Olecranon osteotomy were fixed by tension band Kirschner wires.

A lateral incision approximately 6 to 10 cm was centred over the lateral epicondyle. The elbow joint cavity was open between the extensor carpi ulnaris and the anconeus to reveal capitellum. If the fracture extends to trochlea, the origin of the wrist extensors and lateral collateral ligamentous complex were elevated from the lateral epicondyle to broaden exposure. The lateral joint space was exposed with a medial translation of the elbow joint by exerting a varus‐supination force on it. We use a medial incision of the elbow for cases which a single lateral incision is not enough to expose the fractures.

#### *Reduction and Fixation*

**Fig 2:**
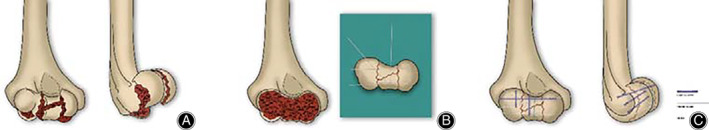
Schematic diagram illustrating the procedures of reduction and fixation. (A) Comminuted coronal shear fracture of distal humerus. (B) Comminuted capitellar and trochlear fracture fragments were taken out of the body and fixed as a whole with Kirschner wires. (C) The comminuted distal humeral coronal shear fracture was fixation with headless screws, Kirschner wires and suture.

Generally it is difficult to reduce Dubberley type 3 fractures in the elbow joint, comminuted capitellum and trochlea fragments can be taken out of the body and fixed as a whole with Kirschner wires and sutures after reduction, and then put into the joint for reduction and fixation with the other distal humerus fragments. Headless screws, Kirschner wires, and Ethicon sutures were used for fixation depending on the complexity of the fracture and stability of the reduction. Lateral collateral ligamentous complex tears and avulsion fracture of epicondyle were repaired primarily with use of drill‐holes and a locking suture technique or suture anchors. Check ligament stability of the elbow after the incision was closed. A diagram was used to illustrate the procedures of reduction and fixation mentioned above (Fig. 2).

### *Postoperative Management*

We regularly use of antibiotics for 24 hours to prevent incision infection. Indomethacin was given orally for heterotopic ossification prophylaxis for 4 weeks. Patients with hinged elbow fixators began active range of motion exercise of the elbow 1 week after operation, while others start from the next day after operation.

Patients were followed up at 1, 2, 3, 6, 9 and 12 months after operation and once every half a year after that. We guided patients to do functional exercise, assess the accuracy of bone union, implants position, and complications from radiography regularly.

### *Outcome Measure*

#### *Clinical Results*

We evaluated clinical results by measuring flexion‐extension arc, forearm rotation arc, MEPS, and the time of fracture union in follow‐up. Protractor was used to measure the flexion‐extension arc of elbow and forearm rotation arc. The time of fracture union was determined by clinical examination, callus growth and the disappearance of the fracture line in X‐ray film in the follow‐up.

#### *Mayo Elbow Performance Score*

Mayo Elbow Performance Score (MEPS) is commonly used to evaluate the function of elbow^11^. The MEPS system includes four parts: pain, motion, stability and elbow function. The aggregate score of MEPS is 100, including 45 points for pain, 20 points for exercise, 10 points for stability, and 25 points for joint function. A score greater than 90 is excellent, 75–89 is good, 60–74 is fair, and a score less than 60 is poor.

#### *Complications*

Postoperative complications, including incision infection, avascular necrosis, internal fixation failure, loosening of Kirschner wire, injury of blood vessel and nerve, instability of the elbow, degenerative osteoarthritis, heterotopic ossification, and removal of implants were recorded and analyzed.

### *Statistical Analysis*

The paired *t*‐test was used to assess the difference between the preoperative and the last follow‐up flexion‐extension arc, forearm rotation arc and MEPS. All patients were divided into three groups according to Dubberley's classification. The flexion‐extension arc, forearm rotation arc, the time of fracture union, and MEPS were compared among three groups. We analyzed the data by SPSS 19.0 (IBM corporation, USA) statistical software. The analysis of variance was used for the comparison among three groups, and Dunnett's test was used for the comparison between two groups. The statistical significance level was set at *P* < 0.05.

## Results

### *Follow‐up*

As a result, all patients were followed up for an average of 17.1 months (range, 12 to 30 months).

### *The Time of Fracture Union*

The average time of fracture union was 8.8 ± 1.9 weeks. The time of fracture union of type 1 fracture (7.7 ± 1.5 weeks) was shorter than that of type 3 (10.3 ± 1.4 weeks) (*P* = 0.024), but there was no significant difference between type 1 and type 2 (8.3 ± 2.0 weeks) (*P* = 0.880) and between type 2 and type 3 (*P* = 0.189).

### *Elbow Flexion‐Extension*

The elbow flexion‐extension arc preoperatively and at the last follow‐up were 87.4° ± 21.7° and 130.5° ± 10.5°, respectively. Compared with preoperative elbow flexion‐extension arc, it significantly increased at the last follow‐up (*t* = 7.79, *P* < 0.001).

At the last follow‐up, the elbow flexion‐extension arc of type 1 fracture (140.0° ± 6.3°) was better than that of type 2 (130.8° ± 3.8°) (*P* = 0.043) and type 3 (122.1° ± 10.7°) (*P* = 0.012), but there was no significant difference between type 2 and type 3 fractures (*P* = 0.209).

### *Forearm Rotation*

The forearm rotation arc preoperatively and at the last follow‐up were 102.6° ± 20.7° and 167.4° ± 6.1°, respectively. Compared with preoperative forearm rotation arc, it significantly increased at the last follow‐up (*t* = 13.09, *P* < 0.001).

At the last follow‐up, the forearm rotation arc of type 1 fracture (172.5° ± 2.7°) was better than that of type 3 (162.1° ± 5.7°) (*P* = 0.006), but there was no significant difference between type 1 and type 2 (168.3° ± 4.1°) (*P* = 0.179) and between type 2 and type 3 (*P* = 0.120).

### *Mayo Elbow Performance Score (MEPS)*

The preoperative MEPS was 60.3 ± 12.3 and the MEPS of the last follow‐up was 85.8 ± 8.5. The MEPS of the last follow‐up was significantly better than preoperative (*t* = 7.43, *P* < 0.001).

**Fig 3:**
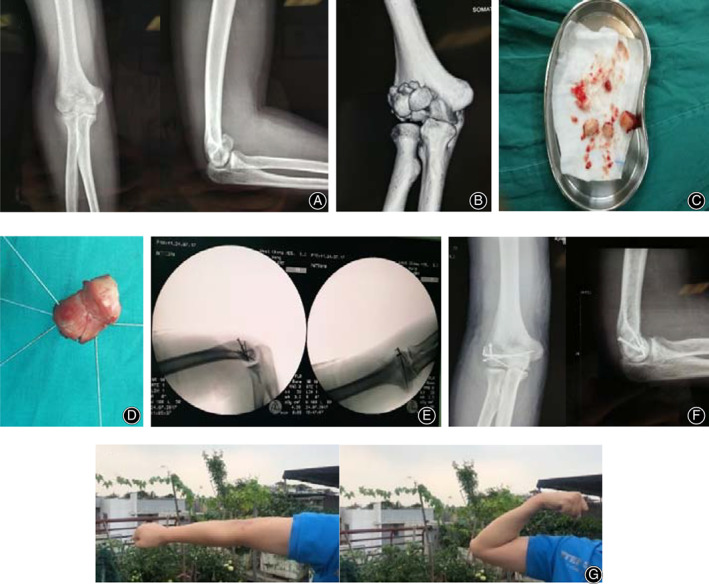
(A) X‐ray films of the fracture. The lateral view showed typical “double arc” sign. (B) Comminuted fracture fragments can be seen in three‐dimensional CT reconstruction. (C) The fracture fragments were taken out of the body. (D) The fragments were fixed as a whole with 1.0mm‐diameter Kirschner wires. (E) X‐ray films after the fracture were fixed with headless screw (DART‐FIRE headless screw produced by WRIGHT company, Memphis, USA) and 1.0mm diameter Kirschner wires. (F) One year after operation, the X‐ray films showed the fracture had healed and the implants were not failure. (G) The flexion and extension images of the elbow 1 year after operation, the MEPS was 90.

**Fig 4:**
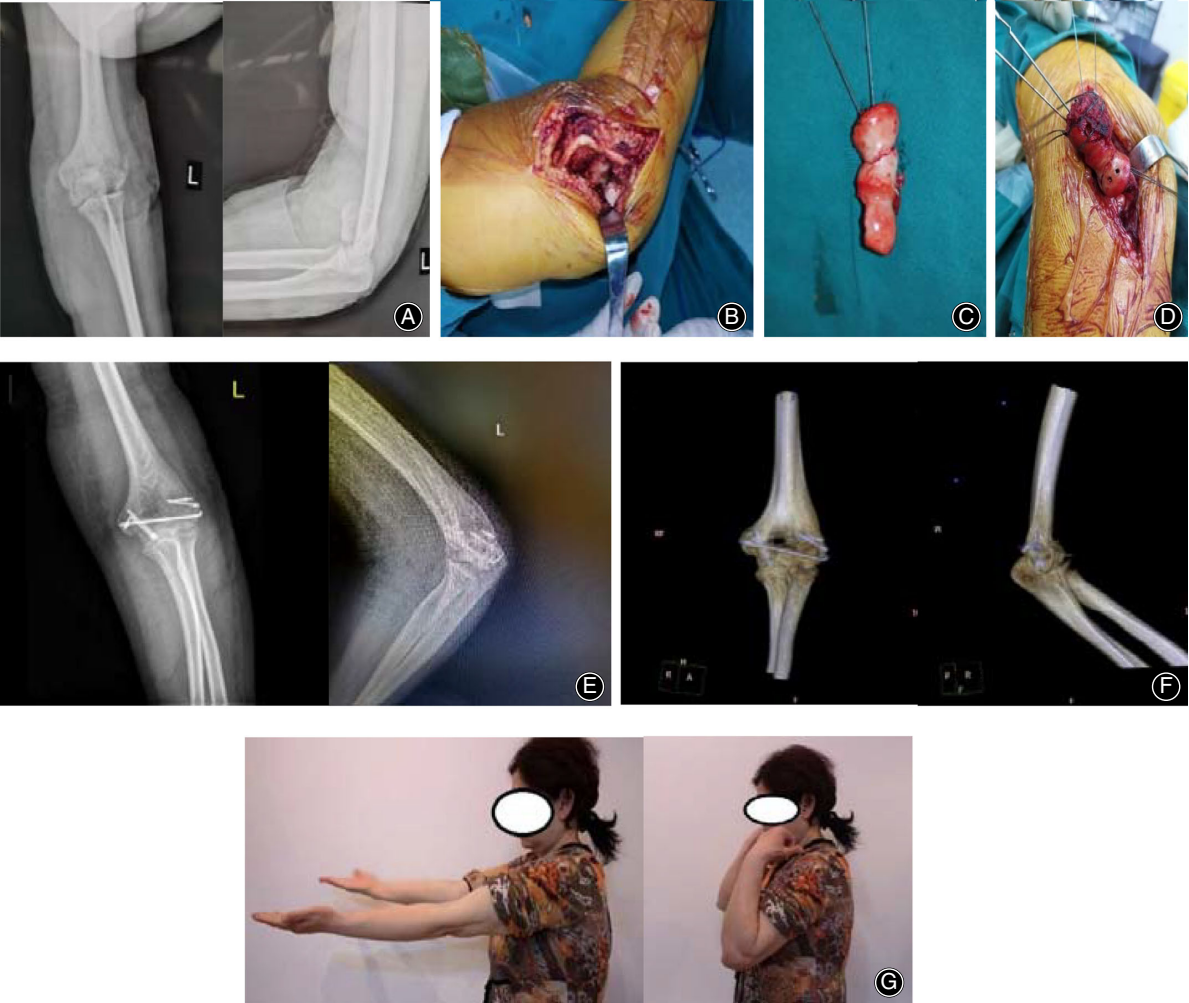
(A) X‐ray imagines showed comminuted fracture of capitellum and trochlea. (B) Trochlear fracture was exposed by additional medial incision. (C) The free fragments were taken out of the body. (D) The fracture fragments were fixed as a whole with Kirschner wires and suture. (E) X‐ray radiographs of the elbow after operation shows three headless screws (DART‐FIRE headless screw produced by WRIGHT company, Memphis, USA), two 1.0mm diameter Kirschner wires and suture were used to fix the fracture. (F) CT with three‐dimensional imagines showed anatomical reduction of fracture and satisfactory implants position. (G) The images of elbow 1 year after operation, the extension was 5° and flexion was 130°. The patient had a good functional elbow outcome with a MEPS was 85.

**Fig 5:**
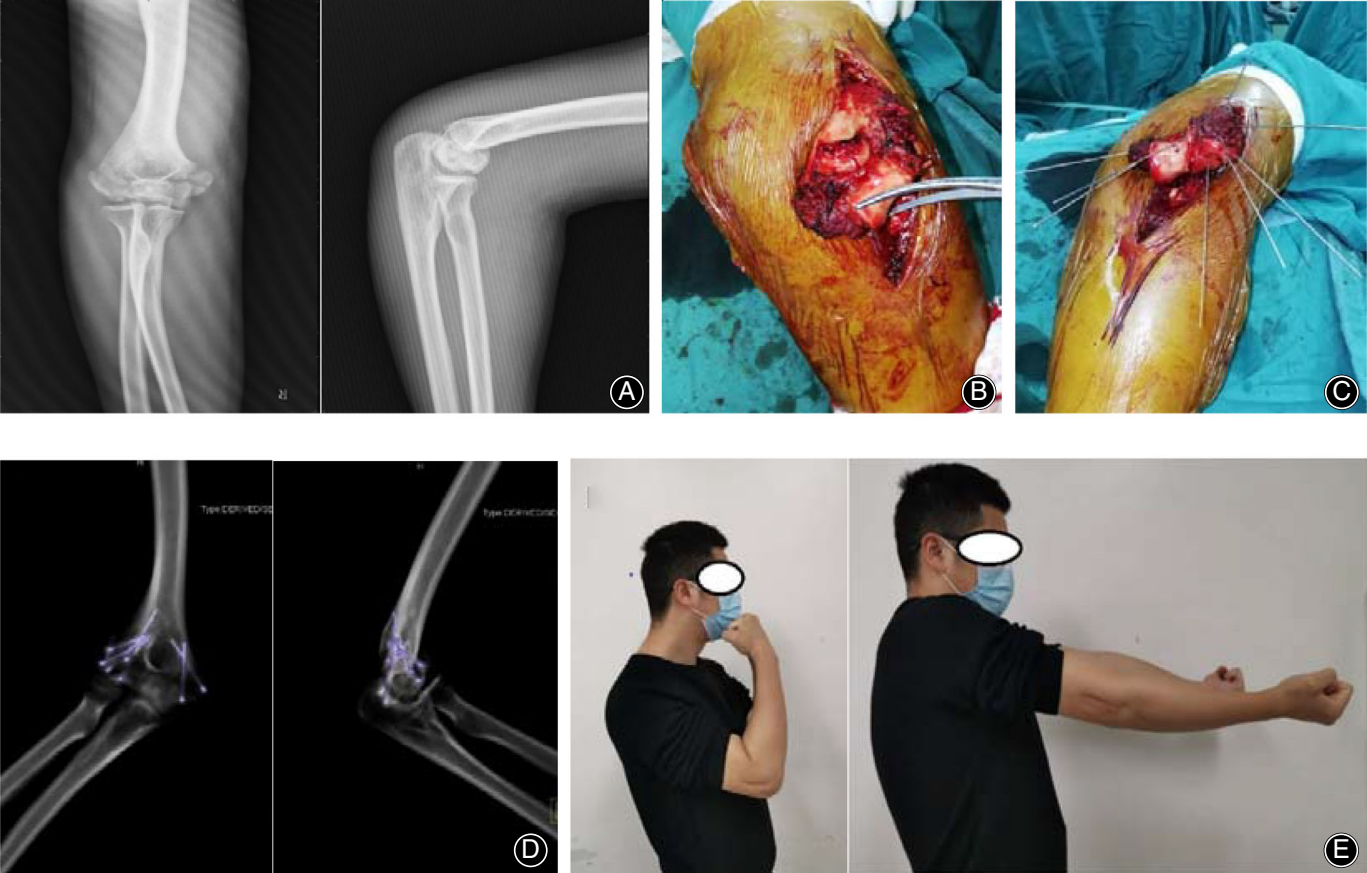
(A) X‐ray films showed comminuted fractures of capitellum and medial epicondyle of humerus. (B) Extended Kocher approach and an additional medial approach were used to exposure the fractures. (C) the fracture fragments were fixed as a whole with Kirschner wires and suture out of the body. (D) X‐ray films of elbow after operation showed five headless screws (DART‐FIRE headless screw produced by WRIGHT company, Memphis, USA) and suture were used to fix the capitellum fracture, and two headless screws were used to fix the medial epicondyle of humerus fracture. (E) One year follow‐up showed that the right elbow function was good. MEPS was 90.

The MEPS of type 1 (94.2 ± 3.8) was better than that of type 2 (86.7 ± 2.6) (*P* = 0.009) and type 3 (77.9 ± 7.6) *(P* = 0.002), but there was no significant difference between type 2 and type 3 (*P* = 0.058). The results were classified as excellent in nine cases, good in eight, and fair in two. The excellent and good rate was 89.5%. See Table 1 for details.Three representative cases are presented in Figs 3–5.

**TABLE 1:** Patient's demography, results and complications

No.	Gender	Age	Side	Type	Treatment	Follow‐up (months)	Flexion/Extension	Pronation / supination	MEPS	Time to union (weeks)	Complication
1	Male	45	Right	1A	Screw	18	145/0	85/90	90	8	‐
2	Female	26	Right	2A	Screw&K wire	12	140/10	80/85	85	8	‐
3	Female	19	Left	3A	Screw&K wire	18	130/10	85/85	75	10	DA*
4	Female	47	Left	1A	Screw	24	145/0	85/90	95	10	‐
5	Male	68	Right	2B	Screw&K wire	24	135/5	80/90	85	12	‐
6	Male	32	Right	1B	Screw&suture	18	130/0	80/90	90	8	‐
7	Male	44	Left	2B	Screw&K wire	12	135/5	85/85	85	6	‐
8	Female	27	Left	3A	Screw&K wire	12	130/10	80/80	75	8	‐
9	Male	29	Right	3A	Screw&K wire	12	140/0	80/90	90	10	‐
10	Female	69	Left	3B	Screw&Kwire&suture&external fixation	18	125/15	75/80	70	12	DA*
11	Male	42	Right	3A	Screw&K wire	24	135/5	80/80	80	10	HO*
12	Female	59	Left	2B	Screw&K wire	30	140/5	85/90	90	8	‐
13	Female	72	Left	3B	Screw&Kwire&suture&external fixation	12	125/15	80/80	70	12	DA*
14	Female	46	Right	2A	Screw&K wire	12	130/5	80/85	85	8	‐
15	Female	55	Left	3A	Screw&Kwire&suture	18	130/5	75/85	85	10	‐
16	Male	38	Left	1A	Screw	18	140/0	80/90	95	6	‐
17	Female	36	Left	1B	Screw	12	140/5	85/85	95	6	‐
18	Male	42	Right	2A	Screw&K wire	12	140/5	80/85	90	8	‐
19	Female	52	Right	1A	Screw	18	145/0	85/90	100	8	‐

DA*, degenerative arthritis; HO*, heterotopic ossification.

### *Complications*

No complications such as incision infection, avascular necrosis, internal fixation failure, loosening of Kirschner wire, injury of blood vessel and nerve, and instability of the elbow joint occurred. There were three cases of degenerative osteoarthritis of the elbow, one case of heterotopic ossification after operation, the elbow function had improved after exercise. A total of 10 patients underwent removal of implants.

## Discussion

### *Options for Internal Fixation*

There is no muscle or ligament attached to the extremely low distal coronal shear fracture fragment, closed reduction is difficult, therefore surgical treatment is necessary in many circumstances. Open reduction and internal fixation can anatomically reduce the fracture and restore the integrity of the joint surface, and rigid fixation has contributed to the early functional exercise of the elbow^5^, ^7^, ^9^, ^12^, ^13^. The main internal fixation methods are headless compression screw, AO cortical screw, Kirschner wire, anti‐slide plate, suture, and so on. Klslowsky reported that the strength of fine‐threaded wires, Herbert screws, and AO screws fixation for Hahn‐Steinthal fracture were better than Kirschner wires in an experimental biomechanical study^14^. Poynton considered that Herbert screw was better than Kirschner wire in the treatment of capitellum frature because compression fixation of fracture allow early postoperative movement, with little damage to articular cartilage and no need to remove implants when buried under the articular surface^15^. Many other authors also recommend headless screw to fix the capitellum and trochlea fractures^13^, ^16^, ^17^, ^18^. Five cases of Dubberley type 1 fracture were fixed with DART‐FIRE headless screws produced by WRIGHT company in our study, Mayo evaluation of elbow was excellent in the follow‐up.

For some extremely comminuted fractures, it is sometimes difficult to use headless screws alone to fix the small fracture fragment, then Kirschner wire can be added^9^, ^19^. In our study, 13 cases were fixed with additional 1.0 mm diameter Kirschner wires. There were no complications such as loosening of Kirschner wire, irritation, or a fracture displacement, but 10 patients underwent removal of implants finally. For some small coronal split fracture fragments which cannot be fixed effectively with screws, if we choose Kirschner wires, the end of the Kirschner wire will face the front of the elbow joint and there was a risk of withdrawing toward the joint cavity, then we can choose suture to bind the fractures. Sodl reported a case of a 12‐year‐old capitellum fracture that achieved satisfactory results, which was fixed by two horizontal mattress sutures placed across the fracture fragment in an “x” configuration^20^. In our study, four cases were fixed with Ethicon sutures and healed smoothly at last.

For some comminuted distal humeral coronal shear fractures which combined with lateral collateral ligamentous complex tear or avulsion fracture of lateral epicondyle of humerus^9^, ^19^, we should repair these structures with drill‐hole and a locking suture technique or suture anchor to restore the stability of the elbow. Giannicola proposed that for severe comminuted fractures which cannot be fixed rigidly, and for those who still have elbow instability after internal fixation and ligament repair, hinged elbow fixators can be used additionally to increase the stability in order to start postoperative functional exercise^21^. In our study, two patients who still have elbow instability after lateral ligament repair were fixed with hinged elbow fixators, and the fixators were usually taken out at 6 weeks after surgery.

### *Surgical Approach Selection*

The Kocher approach of the elbow was used to expose the capitellum fracture between the extensor carpi ulnaris and the anconeus for type 1A fracture. Type 1B and all type 2 fracture need extended Kocher approach. Dubberley recommended that type 3 fractures should be exposed by olecranon osteotomy, which can completely reveal the distal articular surface of humerus, but there are some complications such as nonunion of the osteotomy site and implant irritation^13^, ^22^. Two type 3B fractures of olecranon osteotomy in our study did not have the above complications. We used the lateral extended Kocher approach combined with an additional medial approach to fully expose the entire joint surface in five type 3A fractures and one type 1B fracture combined with a medial epicondyle of humerus fracture, which could ensure the operative field exposure and avoid the risk of complications of olecranon osteotomy.

The comminuted fracture fragments were taken out of the body and fixed into a whole by Kirschner wires and Ethicon sutures. The follow‐up results in these cases showed that nonunion and avascular necrosis of capitellum were not increased with these reduction methods.

### *Prognosis of Different Type Fractures*

In our study we found that the forearm rotation arc of type 1 fracture was better than type 3, and the flexion‐extension arc and MEPS of type 1 fracture were better than type 2 and type 3. Generally speaking, the prognosis was worse as the degree of fracture comminution increased and type 3 fractures are associated with a poorer prognosis. This is consistent with previous reports that the greater the fragmentation of the articular surface, the worse the outcome^4^, ^7^.

### *Analysis of the Complications*

The common complications of extremely low distal humeral coronal plane shear fracture are nonunion, degenerative arthritis, heterotopic ossification, internal fixation failure, elbow stiffness, and avascular necrosis. The fracture healed at an average of 8.8 weeks in our study, and no nonunion or avascular necrosis of capitellum was found. Singh did not discover degenerative arthritis in 14 cases in the follow‐up^16^. In our group, three cases of Broberg‐Morrey grade 1 degenerative arthritis were found, all of which were type 3 fractures^23^. The reason was that there were many comminuted articular fragments, so it was difficult to achieve complete anatomical reduction and rigid fixation. One case had heterotopic ossification 3 months after surgery, which suggested that adequate postoperative drainage of the joint cavity and the use of indomethacin are essential. A total of 10 patients underwent removal of implants finally which suggested that the rate of reoperation using Kirschner wire was greater than other internal fixations.

### *Limitations of the Study*

The level of evidence in our retrospective study is not as high as a prospective randomized control study, the number of cases is relatively small, and the follow‐up time is short. This conclusion is needed to be further confirmed by large‐sample, long‐term follow‐up clinical trials in the future. On the other hand, given the relative infrequency of this condition and the treatment being difficult for comminuted distal humeral coronal plane shear fractures, our retrospective study still has some reference value for surgical treatment of these fractures.

### *Conclusions*

Kocher approach, extended Kocher approach, extended Kocher approach combined with a medial incision and olecranon osteotomy approach are recommended choices for open reduction according to the specific fracture types. Headless screw, Kirschner wire, and suture are useful for the treatment of distal humeral coronal plane shear fractures, which are helpful for anatomical reduction of fractures, restoration of joint integrity, and rigid fixation. The rate of reoperation using Kirschner wire is greater than others. In terms of elbow flexion‐extension arc, forearm rotation arc, fracture healing time and MEPS in the follow‐up, Dubberley type 1 fracture was better than type 2 and type 3 fractures.
